# Spectroscopic Ellipsometry and Correlated Studies of AlGaN-GaN HEMTs Prepared by MOCVD

**DOI:** 10.3390/nano15030165

**Published:** 2025-01-22

**Authors:** Yanlian Yang, Yao Liu, Yaoze Li, Manika Tun Nafisa, Zhe Chuan Feng, Lianshan Wang, Jeffrey Yiin, Lingyu Wan, Benjamin Klein, Ian Ferguson, Wenhong Sun

**Affiliations:** 1 Research Center for Optoelectronic Materials and Devices, Guangxi Key Laboratory for the Relativistic Astrophysics, School of Physical Science & Technology, Guangxi University, Nanning 530004, China2307401031@st.gxu.edu.cn (Y.L.); zfeng6@kennesaw.edu (Z.C.F.); ls-wang@semi.ac.cn (L.W.); 20050059@gxu.edu.cn (L.W.); 2 Southern Polytechnic College of Engineering and Engineering Technology, Kennesaw State University, Marietta, GA 30060, USA; mnafisa@students.kennesaw.edu (M.T.N.); jyiin@kennesaw.edu (J.Y.); bklein8@kennesaw.edu (B.K.); ianf@kennesaw.edu (I.F.); 3 Laboratory of Solid-State Optoelectronic Information Technology, Institute of Semiconductors, Chinese Academy of Sciences (CAS), Beijing 100083, China; 4 State Key Laboratory of Featured Metal Materials and Life-Cycle Safety for Composite Structures, Guangxi University, Nanning 530004, China; 5 Third Generation Semiconductor Industry Research Institute, Guangxi University, Nanning 530004, China; 6 MOE Key Laboratory of New Processing Technology for Nonferrous Metals and the Guangxi Key of Processing for Non-ferrous Metals and Featured Materials, Guangxi University, Nanning 530004, China

**Keywords:** AlGaN/GaN, high-electron-mobility transistor (HEMT), metal–organic chemical vapor deposition, spectroscopic ellipsometry, high-resolution X-ray diffraction, photoluminescence, Raman scattering

## Abstract

A series of AlGaN/GaN high-electron-mobility transistor (HEMT) structures, with an AlN thin buffer, GaN thick layer and Al_0.25_Ga_0.75_N layer (13–104 nm thick), is prepared by metal–organic chemical vapor deposition and investigated via multiple techniques. Spectroscopic ellipsometry (SE) and temperature-dependent measurements and penetrative analyses have achieved significant understanding of these HEMT structures. Bandgaps of AlGaN and GaN are acquired via SE-deduced relationships of refraction index n and extinguish coefficient k vs. wavelength λ in a simple but straightforward way. The optical constants of n and k, and the energy gap E_g_ of AlGaN layers, are found slightly altered with the variation in AlGaN layer thickness. The Urbach energy E_U_ at the AlGaN and GaN layers are deduced. High-resolution X-ray diffraction and calculations determined the extremely low screw dislocation density of 1.6 × 10^8^ cm^−2^. The top AlGaN layer exhibits a tensile stress influenced by the under beneath GaN and its crystalline quality is improved with the increase in thickness. Comparative photoluminescence (PL) experiments using 266 nm and 325 nm two excitations reveal and confirm the 2DEG within the AlGaN-GaN HEMT structures. DUV (266 nm) excitation Raman scattering and calculations acquired carrier concentrations in compatible AlGaN and GaN layers.

## 1. Introduction

Wide-bandgap nitride-based semiconductors, devices and applications have been greatly developed in recent decades [1,2,3], including those based upon oxide semiconductors, such as ZnO [4] and SnO2 [5]. It has been demonstrated that significant power output enhancement from ZnO thin film-based piezoelectric nanogenerators can be achieved via native defect control [4]. Transparent epitaxial SnO2 films with low thermal conductivity and high carrier mobility were developed by domain engineering for thermoelectric power generators [5]. Among nitride-based electronic devices, AlGaN/GaN heterojunction-based high-electron-mobility transistors (HEMTs) have attracted much attention in research and explorations [6,7,8,9,10,11,12,13]. K. Narang et al. [8] pointed that AlGaN/GaN hetero-structures have emerged as an important building block for next-generation high-frequency, high-power devices, due to excellent material properties such as a large energy bandgap, high breakdown field, high peak electron velocity and very high 2DEG concentration superior to its other technological counterparts. J. S. R. Kumar et al. [13] presented a comprehensive review of AlGaN/GaN HEMTs: Architectures and field plate techniques for high-power/high-frequency applications. They are recognized with the superior characteristics mentioned above, suitable for applications for power electronics, microwave systems, communication systems, and so on [6,7,8,9,10,11,12,13]. Research and development (R&D) on AlGaN-GaN HEMTs are currently in the frontier and hot-points of the field, with many journal papers published in the literature during 2024 and some selected ones referred to in [14,15,16,17,18,19].

In AlGaN/GaN HEMT, the two-dimensional electron gas (2DEG) can be generated at the AlGaN/GaN interface with high density and high mobility to achieve a power-switching transistor for next-generation power conversion systems. The AlGaN layer is acting as a barrier layer while GaN is a conductive channel for the generation of 2DEG. AlGaN/GaN HEMTs have been prepared by metal–organic chemical vapor deposition (MOCVD) [8,9,12,14,15,16], molecular beam epitaxy (MBE) [10]. The used substrates include sapphire [13], Si [7,9,12,14,15,16], SiC [8,10,18] and GaN [6]. Some special structural designs were adopted, such as observations of 2DEGs in AlGaN/GaN HEMTs using up-converted photoluminescence excitation [14], with p-NiO gate AlGaN/GaN HEMT [15], using a Ti/Al/Ni/Au (20/150/50/80 nm) ohmic metal stack [16], using the Fe-C co-doped buffer layer and the thinner barrier layer to restrain the degradation caused by RF stress [18], and with Carbon-doped buffer to improve the electrostatic discharge breakdown [19].

In this study, we perform a detailed investigation on a series of AlGaN-GaN HEMT structures with varied AlGaN layer thickness (13–104 nm) grown by MOCVD. They are characterized by variable-angle spectroscopic ellipsometry (VASE), high-resolution X-ray diffraction (HR-XRD), DUV (266 nm) excitation photoluminescence (PL) and Raman scattering, improved with high crystalline structures. Extensive analyses are performed in depth on these HEMT structures, especially for their optical properties influenced by the variation in AlGaN layer thickness. Temperature-dependent (TD) SE measurements were carried out from room temperature (RT) up to near 600 °C, based upon which, three methods in deducing the temperature relationships of the AlGaN and GaN band gaps within HEMTs in the high-temperature range (RT-577 °C) are analyzed comparatively. The validation of our SE-deduced refractive index n and absorption coefficient α of AlGaN-GaN HEMTs, and the deduced Urbach binding energy (E_U_), which is a band tail parameter caused by non-parabolic effects, are further discussed, including the non-parabolicity of the band structure and influences on the refractive index and the absorption coefficient. The HR-XRD measurements and calculations acquired an extremely low dislocation density of 1.6 × 10^8^ cm^−2^ on the GaN layer from our HEMT samples. PL spectral characteristics including the phonon replicas are studied via DUV (266 nm) PL. The two-dimensional electron gas (2DEG) produced in the AlGaN-GaN HEMT structures are investigated via comparative PL experiments under 266 nm and 325 nm excitations. DUV and visible Raman measurements and analyses are used to reveal the layer stress status in HEMTs. The VASE experiments and analyses are employed to obtain the layer thicknesses of HEMT structures, optical constants of refractive index, extension coefficient and absorption coefficient, energy gaps, Urbach energy values (E_U_), and temperature relationships of Eg~T. In comparison with other research works on AlGaN-GaN HEMTs in the literature [6,7,8,9,10,11,12,13,14,15,16,17,18,19], we are aiming to obtain more significant results and deepen the understanding and knowledge of AlGaN-GaN HEMTs.

## 2. Materials and Methods

The experimental sample growth was conducted in a vertical cold wall metal–organic chemical vapor deposition reactor, with two-inch c-sapphire (430 μm thick) used as substrates. Trimethylgallium (TMGa), trimethylgallium (TMAl), and ammonia (NH3) were used as the precursors for Gallium (Ga), Aluminum (Al) and Nitrogen (N) sources, respectively, with high-purity H_2_ as the carrier gas, similarly to the case of Ref. [8] in the literature. Prior to growth, the sapphire substrates were annealed at 1050 °C for 2 min in a hydrogen atmosphere to remove surface contamination and absorb water. Subsequently, a 20 nm thick AlN buffer layer was grown at 750 °C with a V/III ratio of 50,000 under a pressure of 50 torr. Afterwards, the temperature was ramped up to 1040 °C with a V/III ratio of 1400 for the continuous epitaxial growth of ~1.6 μm GaN. Finally, an Al_x_Ga_1−x_N (x~0.25) film with variable thickness of 13–104 nm was grown at the same temperature and with a V/III ratio of 1900. The growth rates of GaN and Al_0.25_Ga_0.75_N are 24 and 26 nm/min, respectively.

**Figure 1:**
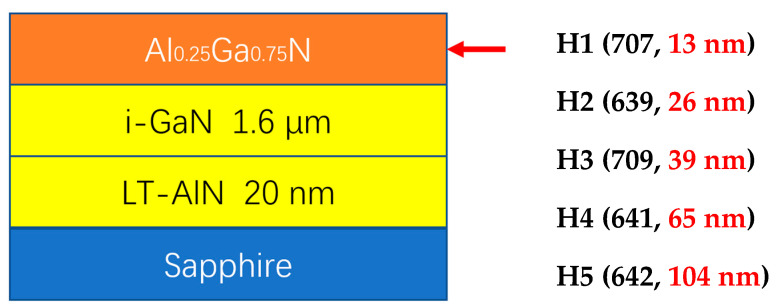
The layer structure of experimental AlGaN-GaN HEMTs.

Figure 1 presents the layer structure of our experimental AlGaN-GaN HEMTs. Five samples are involved, named H1 (707, 13 nm), H2 (639, 26 nm), H3 (709, 39 nm), H4 (641, 65 nm) and H5 (642, 104 nm). The original experiment growth run numbers of 707, 639, 709, 641, and 642, and initially designed Al_0.25_Ga_0.75_N layer thicknesses of 13, 26, 39, 65, and 104 nm, are listed in parentheses “( )” after H-numbers, respectively. It should be noted that from the VASE measurements and simulation analyses in next section, the AlGaN layer thicknesses are determined as 16, 25, 38, 67, and 106 nm for H1-H5, respectively, and the GaN layer thicknesses are about 1556, 1608, 1553, 1600, and 1593 nm, i.e., all near 1.6 μm, for H1-H5, respectively. The practical and real growth rates of GaN and Al_0.25_Ga_0.75_N were adjusted from the designed values, as in the end sentence of last paragraph.

To characterize our HEMTs, variable-angle (VA) spectroscopic ellipsometry (SE), high-resolution X-ray diffraction (HR-XRD), photoluminescence (PL) under deep ultraviolet (DUV) 266 nm and ultraviolet (UV) 325 nm excitations, and Raman scattering under excitations of visible 532 nm and DUV 266 nm have been performed. SE measurements were carried out by using a Mueller matrix ellipsometer, with five incident angles of 50°, 55°, 60°, 65°, and 70°. X-ray diffraction (XRD) experiments were performed by using a PANalytical X’Pert3MRD (Malvern Panalytical). Raman spectra were measured using a Labram HR Evolution (Horiba, London, UK) with a laser wavelength of 532 nm and 325 nm. DUV 266 nm excitation Raman scattering, and photoluminescence (PL) measurements were conducted by using a confocal microscope optical system with wide spectrum from deep ultraviolet to near-infrared, including two lasers of 266 nm and 532 nm, and a spectrometer of iHR550 (Horiba) with gratings of 600 g/mm and 2400 g/mm. All above characterization measurements were conducted at room temperature (RT). Variable temperature (VT) SE measurements were performed in the temperature variation range of room temperature (RT) to 600 °C, with a Linkam temperature stage (THMS600).

From these measurements, the AlGaN-GaN HEMTs are improved with high crystalline structures. More detailed analyses are given in the following sections.

## 3. Results and Discussion

### 3.1. Spectroscopic Ellipsometry Analysis

**Figure 2:**
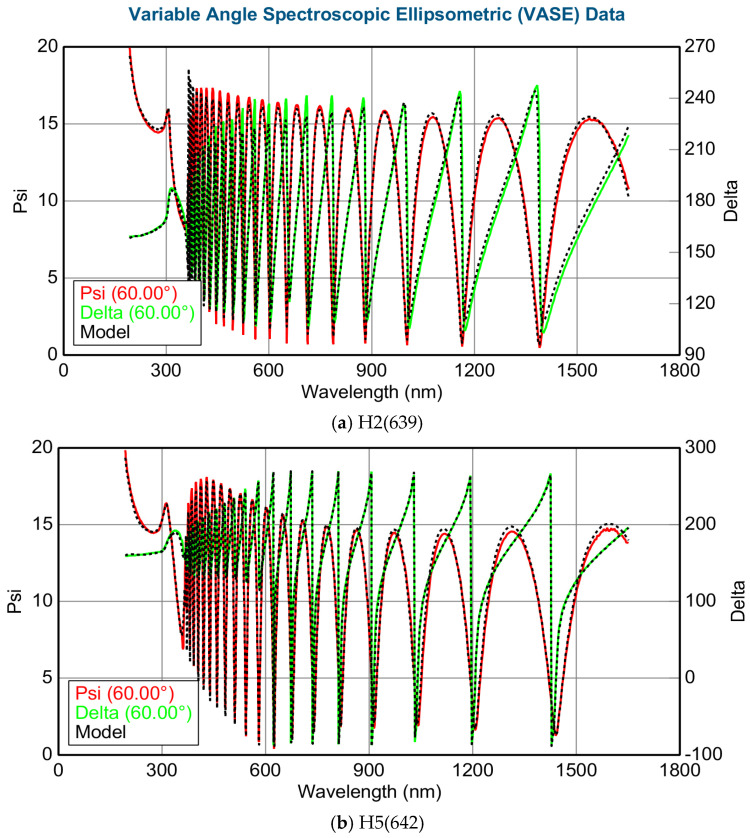
(**a**,**b**) Typical SE Psi (Ψ) and Delta (Δ) spectra at 60° incidence from two samples of H2(639) and H5(642). The experimental SE spectra were measured in the wavelength range of 193–1650 nm, i.e., in the energy range of 6.42 eV down to 0.75 eV.

Variable angle spectroscopic ellipsometry (VASE) measurements were performed for five AlGaN-GaN HEMTs, in the wavelength range of 193–1650 nm, i.e., in the energy range of 6.42 eV down to 0.75 eV, and with five variable incident angles of 50, 55, 60, 65, and 70 degrees, respectively, on each sample. Figure 2a,b show typical SE Psi (Ψ) and Delta (Δ) spectra at 60° incidence from two samples of H2(639) and H5(642). They show oscillation spectra in the wavelength range beyond 360 nm, i.e., below an energy of 3.4 eV, which are due to the optical multi-reflectance from AlGaN/GaN between sapphire substrate and air with an indication of the optical gap of GaN at ~360 nm (~3.4 eV). The sharp peaks at slightly beyond 300 nm roughly indicate the optical gap of AlGaN.

Through simulations on VASE Psi (Ψ) and Delta (Δ) spectra in the wavelength range of 193–1650 nm and with five variable incident angles of 50, 55, 60, 65, and 70 degrees, by using the CompleteEASE software program, the thicknesses and surface roughness values of AlGaN, GaN, and AlN, which are modeled using a Bruggeman effective medium approximation (BEMA) [20] for five AlGaN-GaN HEMTs, were obtained and are listed in Table 1.

**Figure 3:**
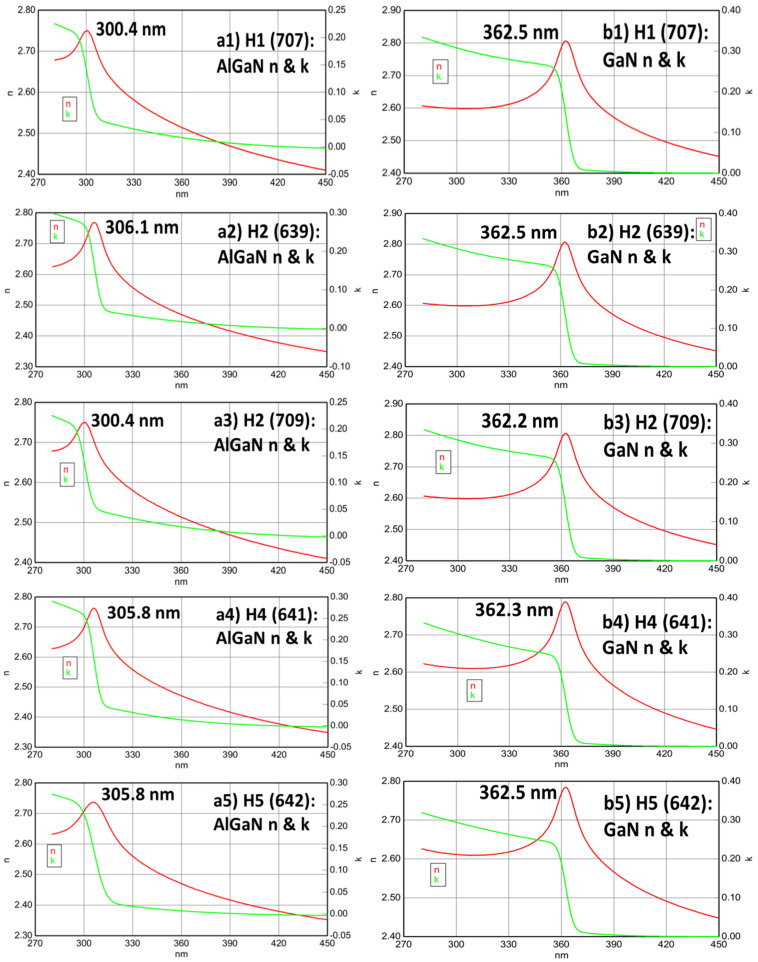
(**a1**–**a5**) and (**b1**–**b5**). Typical graphs of n and k, refractive index and extinction coefficient versus wavelength (nm) from five AlGaN-GaN HEMT samples of H1(707)-H5(642), respectively.

Also, from the SE Psi (Ψ) and Delta (Δ) spectra, and fitting SEsnap files, reflection index, and extinction coefficient (n and k) data versus wavelength (nm) for these five AlGaN-GaN HEMT samples are obtained. To investigate and determine the bandgaps of AlGaN layers and GaN layers within the AlGaN-GaN HEMT structures, Figure 3(a1)–(a5) and (b1)–(b5) exhibit n and k vs. nm in the spectral range of 280–450 nm, i.e., in the energy range of 4.43 eV down to 2.76 eV, in (a1)–(a5) showing the AlGaN layers and in (b1)–(b5) showing the GaN layer, covering both the E_g_(AlGaN) and E_g_(GaN) regions, for the five AlGaN-GaN HEMT samples. Beyond 450 nm, i.e., less than 2.75 eV, all k values are near zero and all n values are gradually decreased, which are not displayed. The maximum peak values in n~λ curve, which corresponds to the middle of the steep dropping k~λ curve, are labeled in all plots. In our recent investigation for MBE-grown GaN/Si with an AlN buffer [21], we have demonstrated the following. From the deduced GaN n~λ curves, the sharp peaks indeed indicate the bandgap of GaN, which are matched well with the middle points of the steep drops from the GaN k~λ curves. These obtained E_g_(GaN) values in units of both wavelength nm and energy eV are listed in Table 1. The five samples have the GaN bandgap energy in 3.415–3.419 eV with a difference within 0.004 eV, i.e., ~0.1%. Similarly, the AlN buffer n~λ curves exhibited sharp peaks with the E_g_ (AlN) in the energy range of 6.274–6.284 eV with a difference within 0.010 eV, <0.2%. Now, we employ this method to label the λ (n_max_) of AlGaN and λ (n_max_) of GaN on their n~λ curves in Figure 3. The bandgap Eg values of AlGaN and GaN layers are acquired and listed in Table 1 (bottom row). Also, we have provided complementary Figures S1 and S2 with the energy (eV) as X-axis units in the complementary information. Photon energies are more natural units when dealing with dispersion models and electronic excitation. To verify and confirm the errors and uncertainty of SE measurements and fits on the layers thicknesses and deduce parameters, we performed comparative cross-section scanning electron microscopy (SEM) measurements on AlN/sapphire structures [20] and other III-nitride structures and determined the accuracy on the scale of ~0.1 nm for thicknesses, which confirmed the peak determination of n(max)~λ curves in Figure 3 and data values in Table 1.

Figure 3(b1–b5) show k~λ curves with a steep drop shape in the narrow spectral range of near 360 nm, which is also true for the absorption coefficient α~λ for GaN [21] because of the relationship of α= 4πk/λ. Similarly, Figure 3(a1–a5) exhibit k~λ curves with steep drops in the short wavelength range of 290–320 nm, indicating the sharp variation in α~λ for AlGaN in five AlGaN-GaN HEMT samples, like α~λ for AlN [20].

**Figure 4:**
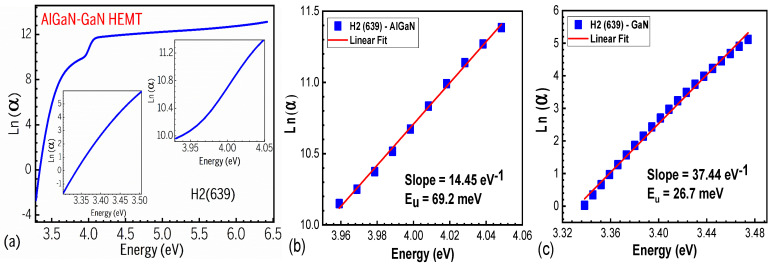
(**a**–**c**). Relationships of Ln(α) vs. energy (eV) for an AlGaN-GaN HEMT sample of H2(639): inside (**a**), two inserts showing the regions around the bandgaps of GaN and AlGaN, respectively; (**b**) determining the Urbach energy E_U_ for the AlGaN layer; and (**c**) determining the E_U_ of the GaN layer, with the slope and E_U_ values marked, respectively.

From the above SE graphs, we plotted the relationships of Ln(α) vs. energy (eV) for an AlGaN/GaN HEMT sample of H2(639), as shown in Figure 4. Two inserts inside (a) are used to determine the Urbach energy E_U_ for AlGaN and GaN layers, from which, at the turning points near Eg of Ln(α) ~eV, the slope is obtained and separately displayed in Figure 4b,c. The Urbach binding energy (E_U_), which is a band tail parameter caused by non-parabolic effects, can be determined from the following formula: 1/Eu=d(lnα)/d(hv) [20]. The obtained values are E_U_(AlGaN) = 69.2 meV and E_U_(GaN) = 26.7 meV, marked in Figure 4b and Figure 4c, respectively, in accordance with Ref. [20].

### 3.2. Temperature-Dependent Spectroscopic Ellipsometry and Further Discussion

**Figure 5:**
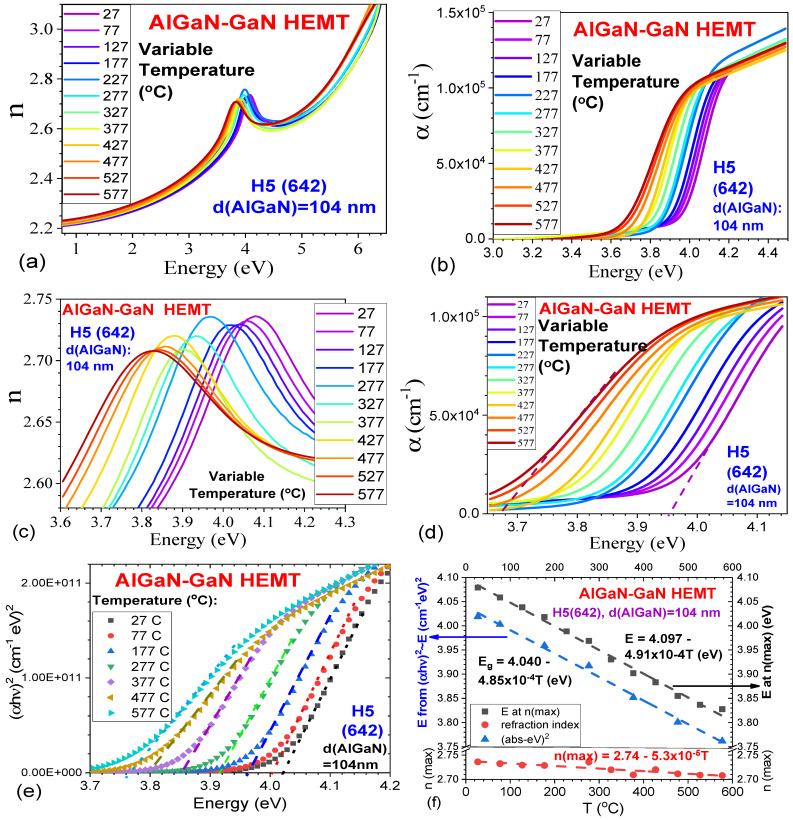
Temperature (27–577 °C) dependences, for an AlGaN-GaN HEMT sample of H5(642), of (**a**) refractive index n vs. energy (eV) in the range of 0.98–6.5 eV, (**b**) absorption coefficient α vs. energy (eV) between 3.0 and 4.5 eV, (**c**) n~eV curves in the narrow energy range of 3.6–4.3 eV, (**d**) absorption coefficient α vs. energy (eV) between 3.6 and 4.15 eV and inserts with two straight lines to obtain Eg at T = 27 °C and 577 °C, (**e**) Tauc plots, i.e., (αhν)^2^ vs. energy (eV) between 3.7 and 4.2 eV, obtaining Eg at seven Ts, and (**f**) temperature relationships of Eg~T from (i) E at n(max) in n~λ curves and (ii) Eg determined by (αhν)^2^ vs. eV in (**e**), as well as (iii) n(max) vs. T from (**c**), respectively.

We have carried out temperature-dependent spectroscopic ellipsometry (TDSE) measurements and analyses. For a typical example of an AlGaN-GaN HEMT sample H5(642), Figure 5 presents its viable temperature (27–577 °C) relationships as follows: (a) the refractive index n vs. energy (eV) in the range of 0.98–6.5 eV, showing n(max) peaks around 4.0 eV, corresponding to the energy gap for the AlGaN layer with x(Al)~0.25; (b) the absorption coefficient α vs. energy (eV) between 3.0 and 4.5 eV, showing the absorption drops from the AlGaN gaps with a clear variation on T; (c) the n~eV curves in the narrow energy range of 3.6–4.3 eV, showing the n(max) peaks and shifts with T clearly; (d) the absorption coefficient α vs. energy (eV) between 3.6 and 4.1 eV with two inserted straight lines to obtain Eg at two Ts; (e) the (αhν)^2^ vs. energy (eV), i.e., Tauc plots, between 3.7 and 4.2 eV, obtaining Eg vs. T; and (f) the temperature relationships of Eg~T from (i) E at n(max) in n~λ curves and (ii) Eg determined by (αhν)^2^ vs eV in (e), as well as (iii) n(max) vs. T from plots at (c), respectively.

Figure 5b shows a clear downward shift in energy eV of the absorption edge, i.e., the bandgap of AlGaN, with the increase in T from 27 °C to 577 °C. Correspondingly, from Figure 5c, the n(max) peaks in the n~λ curves are shifted downwards on the energy with the T-increase from 27 °C to 577 °C. As shown in Figure 5f, the Eg obtained from (αhν)^2^ vs energy (eV), i.e., Tauc plots in Figure 5e, obeys a linear expression on T: Eg = 4.040–4.85 × 10^−4^T. Also, the EPL at n(max) from the n~λ curves in Figure 5c obeys another linear expression on T: EPL = 4.097–4.91 × 10^−4^T; however, their values are about 0.5 eV higher than the Eg values determined from (αhν)^2^ ~ eV, i.e., Tauc plots. In Figure 5d for the α ~ eV curves, from the linear part, extending a straight line to reach the α = 0 axis, we can obtain E = 3.95 eV for T = 27 °C and E = 3.67 eV at T = 577 °C, respectively. Both values are about 0.8 eV lower than the corresponding values obtained from the (αhν)^2^~eV relationship at two temperatures. In a comparison of the three methods in determining the bandgap values of AlGaN in AlGaN-GaN HEMT samples, it is seen that the Eg determined from the n(max) point in the n~λ curve is a little higher than that obtained from the (αhν)^2^~eV relationship, and that the Eg determined from the α~eV curve is a little lower. But both simple methods can be used for rough estimation, which is useful for the quick characterization in industry production.

The n(max)~T relationship has also a linear expression on T: n(max) = 2.74–5.3 × 10^−5^T. Previously, M. Baeumler et al. [22] performed SE assessment of layer composition and thickness in AlGaN/GaN HEMT structures, in which x(Al) = 36.9% and Eg(AlGaN) = 4.2193 eV. But it is still scarcely seen using SE technology in the investigation of AlGaN-GaN HEMTs.

In the literature, to study the optical characteristics of AlGaN-GaN HEMTs, researchers usually employed photoluminescence (PL) [19,23,24,25,26,27,28] and electroluminescence (EL) [14,18]. D. M. Tobaldi et al. [26] studied GaN/AlGaN/GaN heterostructure grown by RP-MOCVD on 4H-SiC substrate, by PL excited with a He−Ag laser at 224.3 nm (5.5 eV) over 10–300 K, showing a near-linear PL peak ~ T relationship in the range of 200–300 K. Also, S. Vitusevich et al. [27] performed the PL of AlGaN-GaN HEMT under the 325 nm excitation over 80–480 K, exhibiting a near-linear PL peak ~ T relationship in the 200–480 K range. The linear relationships presented in Figure 5f from our SE measurements and analyses are matched with other results from the PL experiments reported above [26,27]. Summarizing Section 3.3, the VASE experiments and analyses helped to precisely obtain the layer thicknesses of HEMT structures, the refractive index, the Urbach energy values (E_U_), and the temperature relationships of Eg~T.

In addition, we would like to confirm the validation of our SE-deduced refractive index n and absorption coefficient α of AlGaN-GaN HEMTs: In particular, M. Baeumler et al. [22] studied AlGaN/GaN HEMT structures by spectroscopic ellipsometry and presented, in their Figure 3 and Figure 5, the real dielectric constant <ε1> peak values of near 8, i.e., n~2.8, which matches our n(max) values in our Figure 5a. Recently, G. Baldi et al. [29], employed the broadband spectroscopic ellipsometry to investigate complex GaN/AGaN/AlN film stacks, also showing the n(max) values of ~2.8 (their Figure 5). For the absorption coefficient α of AlGaN-GaN HEMTs, due to the lack of SE studies in the literature, we compare our α values in our Figure 5b with the AlGaN α values reported in Figure 10 by Y. Liu et al. [30], the relations of α^2^~eV over 300 K–823 K are shown. Our relations of α~eV over 27–577 °C, i.e., 300 K–850 K in Figure 5b, are matched with them.

Furthermore, we would like to discuss the non-parabolicity of the band structure and influences on the refractive index and the absorption coefficient. The refractive index is related to the dielectric function of the material, and non-parabolicity can cause a shift in the effective mass of the carriers, which influences the material’s dielectric properties and thus alters the refractive index. Non-parabolicity affects both the refractive index and the absorption coefficient by modifying the electronic structure of the material. It leads to changes in the density of states, effective mass of charge carriers, and the transition probabilities for optical processes. As a result, both linear and nonlinear optical properties are influenced by the non-parabolic nature of the band structure. Z.-H. Zhang et al. [31] investigated the effect of conduction band non-parabolicity on the nonlinear optical properties in GaAs/Ga_1−x_Al_x_As quantum wells, but there is a lack of similar studies on AlGaN/GaN heterostructures, which is worth exploring.

### 3.3. High-Resolution X-Ray Diffraction Analysis

**Figure 6:**
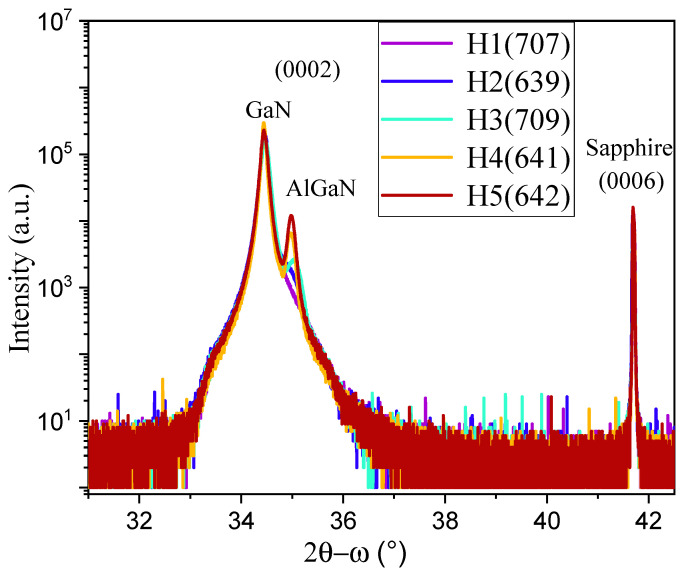
HR-XRD scans of five AlGaN-GaN HEMTs of H1(707), H2(639), H3(709), H4(641), and H5(642), respectively, with the substrate sapphire (0006) peak at 41.70°. The first-order GaN (0002) peaks are located at ~34.5° and AlGaN (0002) are at 35.0°–35.1°.

Figure 6 shows the high-resolution X-ray diffraction (HR-XRD) scans of five AlGaN-GaN HEMTs in 2θ between 32° and 42.5°. The substrate sapphire (0006) peak at 41.70° served as the calibration of all XRD scan spectra. The first-order GaN (0002) and AlGaN (0002) XRD peaks are observed in the 2θ range of 34.2°–35.4°, with the GaN (0002) peaks located at ~34.5° and AlGaN (0002) at 35.0°–35.1°.

**Figure 7:**
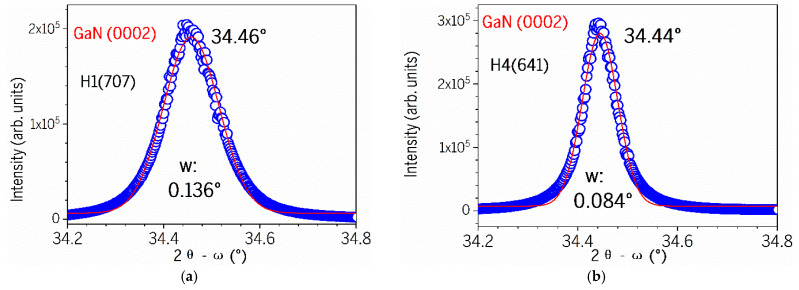

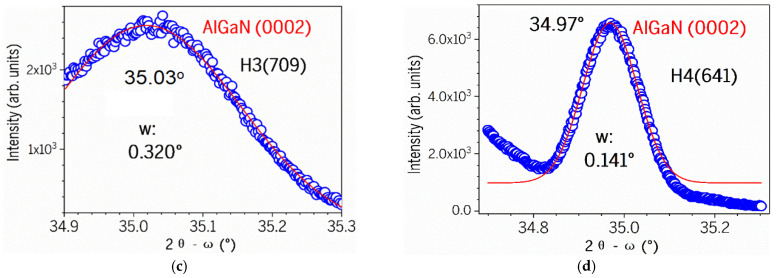
Typical Gaussian fittings of GaN (0002) for (**a**) H1 and (**b**) H4, and AlGaN (0002) for (**c**) H3 and (**d**) H4, with fitted parameters listed in each graphs, respectively.

The Gaussian fits on five GaN (0002) peaks and three AlGaN (0002) peaks have been performed. Figure 7 shows the typical fitting curves of H1 and H4 for GaN (0002), and H3 and H4 for AlGaN (0002), respectively. The data that were used for calculating the screw dislocation densities are listed in Table 2.

To analyze the structural features of five AlGaN-GaN HEMT samples and compare their crystal characteristics, further quantitative calculations are performed below. Referring to [7] for the AlGaN/GaN heterojunctions, the screw dislocation density can be calculated according to the full width at half-maximum (FWHM) of XRD peak, in the following formula:

*D_screw_* = *β*^2^/(4.36*b*^2^) (1)

where *D_screw_* is the screw dislocation density, *β* is the FWHM value of the (0002) XRD peak, and *b* is the Burgers vector. Following the method used by Z. C. Feng et al. [21] for MBE-grown GaN on Si, the screw dislocation density *D_screw_* of five GaN films is calculated accordingly and listed in Table 2. It is found that the sample H4(641) possesses the narrowest GaN FWHM and lowest dislocation density of 1.6 × 10^8^ cm^−2^, H5(642) has the second-lowest *D_screw_* of 3.7 × 10^8^ cm^−2^, while H3(709) exhibits the worst density. Our lowest obtained dislocation density of 1.6 × 10^8^ cm^−2^ is better than values of AlGaN-GaN HEMTs reported in the literature [7,10].

For the case of AlN, W. Wei et al. [32] reports that the AlN Burgers vector lengths of screw type and edge type TDs are b_screw_ = 0.4982 nm and b_edge_ = 0.3112 nm, respectively. All our five AlGaN-GaN HEMTs possess an x(Al) of near 0.25. So, it is reasonable for us to take the linear insert for the Burgers vector length of b_screw_(Al_0.25_Ga_0.75_N) between b_screw_(GaN) and b_screw_(AlN), i.e.,

b_screw_(Al_0.25_Ga_0.75_N) = b_screw_(AlN) + 0.25{b_screw_(GaN) − b_screw_(AlN)} = 0.4982 + 0.25 × {0.5185 − 0.4982} = 0.5033 (nm) (2)

So, according to the XRD data and fitting results of AlGaN (0002) peaks, we can obtain, for AlGaN, b = 0.5033 × 10^−7^ cm, b^2^ = 0.2533 × 10^−14^, and 4.36b^2^ = 1.104 × 10^−14^ cm^2^. The calculation results of screw dislocation densities of the three AlGaN films are listed in Table 3.

It can be seen that sample H5(642) possesses the narrowest AlGaN FWHM and lowest dislocation density of 4.66 × 10^8^ cm^−2^, H4(641) has the second-lowest *D_screw_* of 6.93 × 10^8^ cm^−2^, and H3(709) has a much larger *D_screw_*.

### 3.4. Photoluminescence Analysis

**Figure 8:**
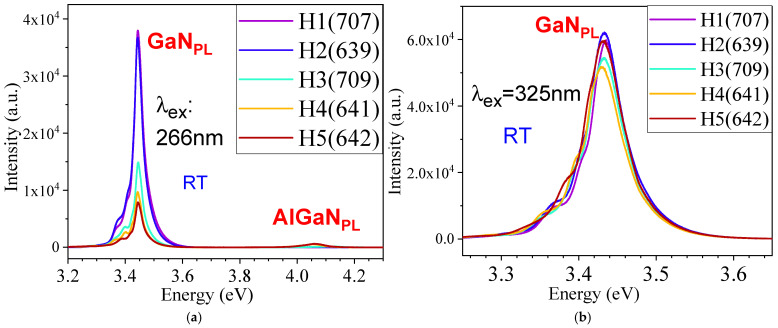
RT PL spectra of five AlGaN-GaN HEMTs of H1(707), H2(639), H3(709), H4(641), and H5(642), (**a**) under excitation of 266 nm with GaN_PL_ peaks at ~3.45 eV and AlGaN_PL_ peaks located between 4.05 and 4.15 eV, (**b**) under excitation of 325 nm with GaN_PL_ peaks at ~3.43 eV, respectively.

Figure 8 presents the RT photoluminescence (PL) spectra under excitations of (a) 266 nm and (b) 325 nm, respectively, for five AlGaN-GaN HEMTs.

**Figure 9:**
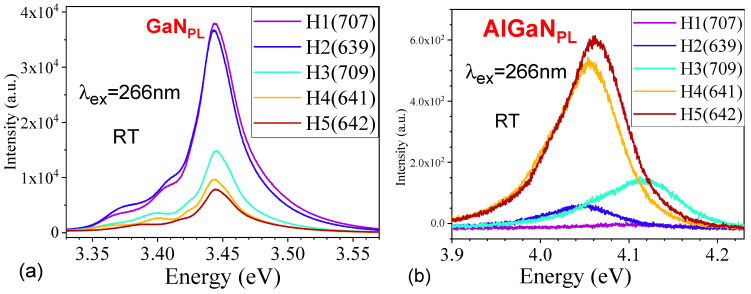
Expanded PL spectra (ex: 266 nm) of (**a**) GaN and (**b**) AlGaN bands for five AlGaN-GaN HEMTs of H1(707), H2(639), H3(709), H4(641), and H5(642), respectively.

Figure 9 exhibits the expanded PL (ex: 266 nm) graphs of (a) GaN_PL_ and (b) AlGaN_PL_ bands for five AlGaN-GaN HEMTs. The Lorentz fits on these PL curves were performed.

**Figure 10:**
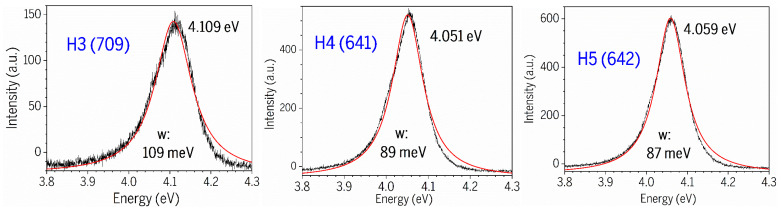
Lorentz fits on PL spectra (ex: 266 nm) of AlGaN PL bands from five AlGaN-GaN HEMTs.

Figure 10 exhibits the Lorentz fits on the PL spectra (ex: 266 nm) of AlGaN bands for three AlGaN-GaN HEMTs, with fitted PL peak and FWHM values marked in the graphs and listed in Table 4, including H1(707) and H2(639). It can be seen that H3(709) has its AlGaN PL peak near 4.11 eV, slightly higher than the other four HEMTs, which is due to the fact, from HR-XRD measurements on five AlGaN-GaN HEMPTs in Figure 6 and the insert, that H3(709) possesses its AlGaN (0002) peak at ~35.03°, higher than those peaks at slightly below 35.00° from H1(707) and H4(641), as well as H2(639)/H6(642) with the AlGaN (0002) peaks as shoulders of GaN (0002) only.

**Figure 11:**
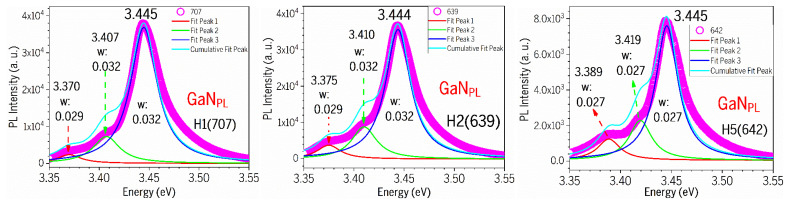
Lorentz fits on PL spectra (ex: 266 nm) of GaN PL main leak and side emission bands from three AlGaN-GaN HEMTs.

Figure 11 presents the Lorentz fits on the PL spectra (ex: 266 nm) of the GaN PL main leak and side emission bands from three AlGaN-GaN HEMTs. Table 4 lists the values of the GaN PL main leak and side emission bands, and their FWHMs, followed the values of the AlGaN ones, including H3(709) and H4(641).

From a sample H1 (707), two side PL bands are located below the GaN main peak (ex: 266 nm) with difference of about 75 meV and 38 meV. Sample H2 (639) has two side PL bands located below the GaN main peak (ex: 266 nm) with a difference of about 69 meV and 34 meV. The GaN has an E_2_(high) frequency of 568 cm^−1^, i.e., 70.4 meV and an A_1_(LO) of 735 cm^−1^, i.e., 91.1 meV. So, H2 (639) possesses the E2(high) phonon replica, and other side band emissions might be due to some impurities unknown yet.

The GaN_PL_ main peak under 266 nm excitation is located at ~3.45 eV in Figure 9a, higher than the ordinary GaN emission of 3.42 eV at RT [19,27], and higher than the GaN_PL_ peak of ~3.43 eV under 325 nm excitation in Figure 8b. This difference of ~20 meV may be caused by the free electron carriers inserted from the top AlGaN layer, forming the two-dimensional electron gas (2DEG) in the interface of AlGaN/GaN. The difference of ~20 meV in the GaN_PL_ peak between two excitations of 266 nm and 325 nm can be explained as follows. The 325 nm (3.8 eV) laser light goes through the AlGaN layer, unable to induce the transitions between the conduction band and valence band of AlGaN layer with x(Al)~0.25 and E_g_(AlGaN)~4 eV, but excites the transitions between the conduction band and valence band of GaN layer, producing the pure GaN_PL_ emission at ~3.43 eV only. However, as they are using the 266 nm (4.66 eV) excitation, both AlGaN and GaN layers can be excited, causing some electron carriers from AlGaN layer entered GaN layer to contribute to the PL transitions at the GaN_PL_ bands, leading to the GaN_PL_ band under 266 nm excitation being higher in energy than the GaN_PL_ band under 325 nm excitation. So, our comparative PL experiments under 266 nm and 325 nm two excitations reveal and confirm the existence and influence of 2DEG within the AlGaN-GaN HEMT structures.

Previously, Kim et al. [23] studied the low-T PL characteristics of AlGaN/GaN heterostructure and assigned strong broad emissions (12K) located at approximately 3.39–3.45 eV and the related phonon replicas below the GaN bandgap due to 2DEG. D. Jana et al. [24] performed low-T (10K) PL on AlGaN/GaN HEMTs, observed the GaN-related excitonic features, DAP, and their phonon replicas plus some fine oscillations, and assigned some broad emissions with these fine oscillations to the 2DEG states located below the GaN fundamental bandgap of 3.49 eV (10K) by about 0.1 eV. Recently, Y.-T. Chen et al. [14] investigated the up-converted photoluminescence excitation (UPLE) spectra (RT) of AlGaN/GaN HEMT and attributed the 3.335-eV excitation peak to the 2DEG, which is located about 0.1 eV below the GaN fundamental edge emission. Also, a broad peak around 3.75 eV was observed, which is associated with the band-edge emission of the AlGaN layer. Accordingly, our GaN_sidePL2_ peak observed at 3.37 eV in Figure 11 might possibly be due to the 2DEG in our AlGaN-GaN HEMTs.

Reviewing other works of photoluminescence (PL) and temperature-dependent (TD) PL studies on AlGaN-GaN HEMTs in the literature [15,19,25,26,27,28], M. A. Munshi et al. [19] studied the GaN 3.4 eV PL peak and defect yellow band, and S. Vitusevich et al. [27] performed the VT (80–480 K) PL under 325 nm excitation. These [19,27] and some other uncited works performed PL from AlGaN-GaN HEMTs but detected mainly only luminescence from GaN. Beyond these [19], W. Yao et al. [25] used a He–Cd laser with an excitation wavelength of 175 nm, detected PL at 50, 100 and 300 K, and reported PL bands of 3.907, 3.89, and 3.863 eV from the top AlGaN layer. D. M. Tobaldi et al. [26] employed a He−Ag laser operating at 224.3 nm (5.5 eV) for PL studies over 77–300 K and detected the 77 K Pl spectrum consisting of the dominating donor-bound exciton emission band with a peak centered at 3.48 eV. Y. Peng et al. [24] employed the 261 nm excitation to probe the p-NiO/AlGaN/GaN heterostructure with PL bands at 330 nm (3.75 eV) from AlGaN and 365 nm (3.40 eV) from GaN. C.-F. Lin et al. [28] used a 266 nm laser to excite the PL signals including GaN 362 nm (3.425 eV) and AlGaN 328 nm (3.780 eV). These predicate that it is needed to continually enhance the penetrative PL studies on AlGaN-GaN HEMTs. Our TDSE studies in RT to near 600 °C in Figure 5 provide complementary temperature dependence characteristics of AlGaN-GaN HEMTs, which are also worth further investigating.

### 3.5. Visible (52 nm) and DUV (266 nm) Raman Spectroscopy Measurements

**Figure 12:**
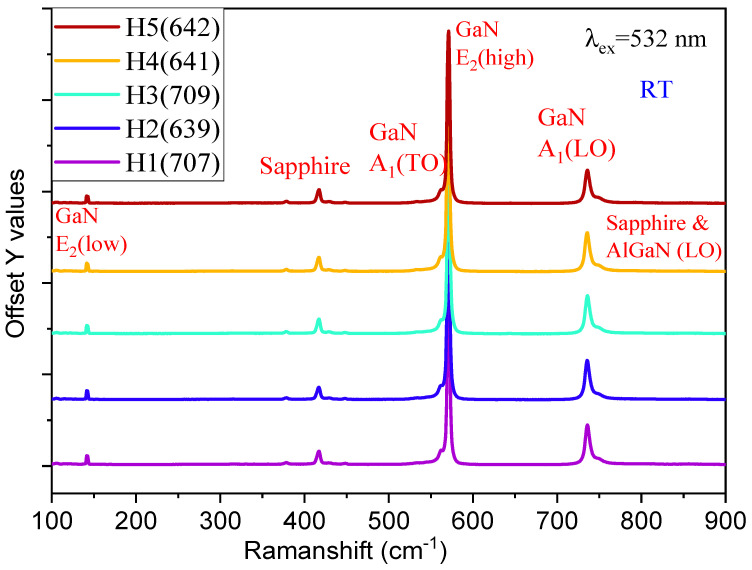
Visible 532 nm excitation spectra of five AlGaN-GaN HEMTs of H1(707), H2(639), H3(709), H4(641), and H5(642), respectively.

Visible Raman scattering measurements were first performed using 532 nm laser excitation for 5 HEMT samples, as shown in Figure 12.

These Raman spectra of 5 AlGaN-GaN HEMT/sapphire samples exhibit the dominant GaN E_2_(high) phonon modes {all spectra normalized in intensity to this peak} plus the left shoulders for GaN A_1_(TO), the second strongest GaN A_1_(LO) at ~735 cm^−1^ plus the right shoulders for mixed sapphire and AlGaN LO, GaN E_2_(low) at about 140 cm^−1^, and sapphire modes at ~420 and 378 cm^−1^ [33]. The above Raman mode intensities reflect the fact that all samples possess about 1.6 μm thick GaN layer, much thicker than the AlGaN layer (< 104 nm).

We have practiced performing UV (325 nm) excitation Raman measurements on these five samples. The GaN E_2_(high) modes plus the right shoulders for AlGaN E_2_(high) modes, and GaN E_2_(low) modes plus the right shoulders for AlGaN E_2_(low) modes, can be recognized weakly, but all emerged in the low-energy side tails of the strong GaN 3.4 eV luminescent bands, still not clearly exhibited.

**Figure 13:**
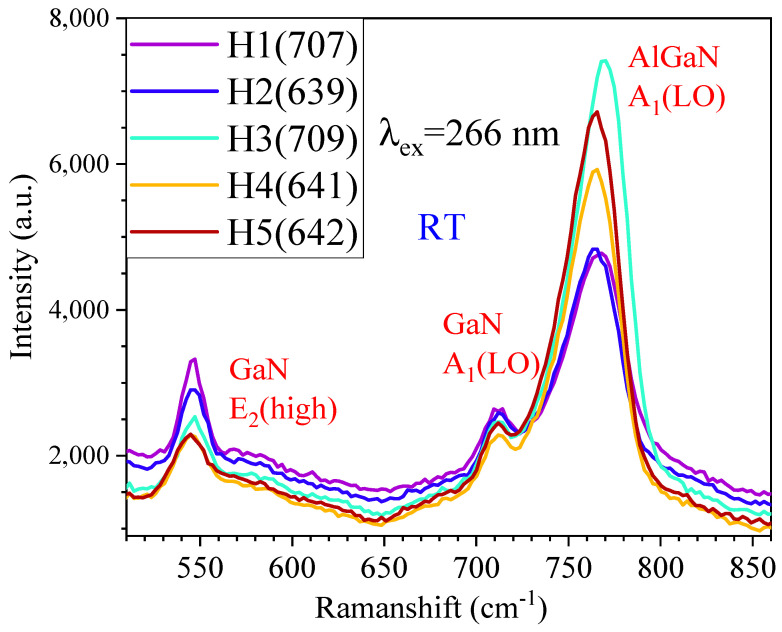
DUV 266 nm excitation Raman spectra of five AlGaN-GaN HEMTs of H1(707), H2(639), H3(709), H4(641), and H5(642), respectively.

We performed the DUV (266 nm) excitation Raman measurements on these five samples, with the same absolute intensity count as shown in Figure 13, and the strongest Raman mode was AlGaN A_1_(LO), while the GaN E_2_(high) mode is the second-strongest one. The GaN A_1_(LO) mode stands weaker in the left side of AlGaN A_1_(LO), while AlGaN E_2_(high) mode emerged in the right side of GaN E_2_(high) weakly. The GaN E2(high) mode has its peak intensity decreased with the increase in the top AlGaN layer thickness (see Figure 13) due to the absorption of the top AlGaN layer to the incident 266 nm laser light. It can be seen that the two thinnest AlGaN films of 707 and 639 (i.e., H1 and H2) have the weakest AlGaN A1(LO) peaks, while the film of 709, i.e., H3, with a mid-thickness of AlGaN, possesses the strongest AlGaN A1(LO) peak, which indicates that this sample has superior quality compared with the others.

### 3.6. Raman Spectroscopy Simulations

Our five AlGaN-GaN HEMT samples possess about 1.6 μm thick GaN layers. By way of spatial correlation model (SCM) analyses on GaN E_2_(high) modes, the Raman spectral intensity, which is characteristic of GaN layer quality, can be presented as follows:

(3) Iω∝∫01exp−q2L24d3qω−ωq2+Γ0/22

where q is expressed in units of 2π/a, a is the lattice constant, and *L* is the correlation length in units of a, representing the phonon propagation length and characterizing the crystalline perfection of the material. The dispersion relation for optical phonons can be represented by an analytical form:

ω^2^(q) = A + {A^2^ − B [1 − con(πq)]}^1/2^, (4)

or ω(q) = A − Bq^2^ (5)

where A and B are adjustable parameters [33]. This spatial correlation model (SCM) has been employed by the author and collaborators to analyze many semiconductors and oxides, such as SiC, GaN, AlN, and AlGaN [33].

**Figure 14:**
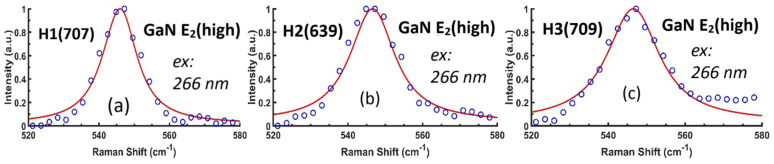
Experimental and fitted DUV 266 nm Raman spectra of GaN E_2_(high) modes from three AlGaN-GaN HEMTs, (**a**) for H1(707), (**b**) for H2(639) and (**c**) for H3(709), respectively, with GaN E_2_(high) peak, and calculated parameters listed in Table 5.

Figure 14 exhibits the DUV 266 nm excitation Raman spectral information of GaN E_2_(high) modes, with experimental data in open symbols and SCM fits by red lines, for three AlGaN-GaN HEMT samples. The calculated parameters based upon SCM are listed in Table 5, including H4(641) and H5(642). It is seen that the correlation length L values from these five AlGaN-GaN HEMT samples are comparable with the best values reported in the literature [21,33].

It can be seen that the GaN E_2_(high) peak is located at near 546 cm^−1^, ~22 cm^−1^ lower than the stress-free GaN E_2_(high) peak at 568 cm^−1^, indicating that the near-top GaN layer is under heavy tensile stress in the area less than 200 nm.

**Figure 15:**
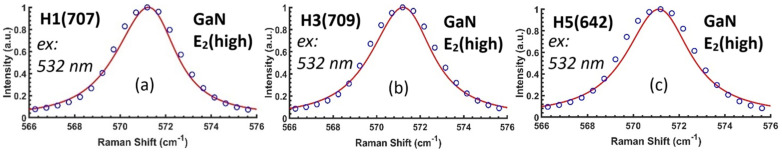
Experimental and fitted visible 532 nm Raman spectra of GaN E_2_(high) modes from three AlGaN-GaN HEMTs, (**a**) for H1(707), (**b**) for H3(709) and (**c**) for H5(642), respectively, with GaN E_2_(high) peak, and calculated parameters listed in Table 6.

It is interesting to compare the above values under DUV 266 nm excitation with the 532 nm Raman spectra of GaN E_2_(high) modes from three AlGaN-GaN HEMTs, as shown in Figure 15. The GaN E_2_(high) peak, and calculated parameters for them are listed in Table 6, including H2(639) and H4(641).

It is found that the GaN E_2_(high) peak is located at near 571 cm^−1^, ~3 cm^−1^ higher than the stress-free GaN E_2_(high) peak at 568 cm^−1^, indicating that the whole 1.6 μm thick GaN layer is under compressive stress. But they have a correlation length L that is much longer than that under 266 nm detection, and the damping constant is much smaller than that under 266 nm detection.

Also, the E_2_(high) peak measured under 266 nm excitation is located at ~546 cm^−1^, which is lower than the E_2_(high) value of 568 cm^−1^ from stress-free GaN [32]. Because the penetration depth of the 266 nm into AlGaN and GaN is about 200 nm, and our experimental AlGaN-GaN HEMT samples have an AlGaN layer thicknesses of 13–104 nm, the 266nm laser light probes only the top area of the 1.6 μm thick GaN layer, which is under tensile stress. It is interesting to find from Table 5, for a correlation length L of the GaN E2(high) mode, the 266 nm excitation Raman spectra were calculated as 19-21-20-27-26 Å, and from Table 4, for a correlation length L of GaN E2(high) mode, the 532 nm excitation Raman spectra were calculated as 27-29-30-31-37 Å. These indicate that the quality of the GaN layer in the GaN-AlGaN HEMT structure is improved with the increase in the top AlGaN layer thickness.

From theoretical analyses and calculations in Raman LO mode, the carrier concentrations can be obtained. Raman scattering can offer a non-destructive experimental technique for the determination of the carrier concentration due to doping in semiconductors through LO phonon and plasma coupling (LOPC). A set of formulas for LOPC were developed to determine the free carrier concentration in these wide-bandgap semiconductors. The Raman intensity of LOPC mode can be expressed as [32] follows:

(6) $$I_{LOPC}={\left.\frac{d^{2}S}{d\omega d\Omega }\right|}_{A}=\frac{16\pi hn_{2}}{V_{0}^{2}n_{1}}\frac{\omega_{2}^{4}}{C^{4}}\left(\frac{d\alpha }{dE}\right)\left(n_{\infty }+1\right)AIm\left(-\frac{1}{\varepsilon }\right)$$

where

$$A=1+2C\frac{\omega_{T}^{2}}{\Delta }\left[\omega_{p}^{2}\gamma \left(\omega_{T}^{2}-\omega^{2}\right)-\omega^{2}\eta \left(\omega^{2}+\gamma^{2}-\omega_{p}^{2}\right)\right]+C^{2}\left(\frac{\omega_{T}^{4}}{\Delta \left(\omega_{L}^{2}-\omega_{T}^{2}\right)}\right)$$

(7) $$\times \left\{\omega_{p}^{2}\left[\gamma \left(\omega_{L}^{2}-\omega_{T}^{2}\right)+\eta \left(\omega_{p}^{2}-2\omega^{2}\right)\right]+\omega^{2}\eta \left(\omega^{2}+\gamma^{2}\right)\right\}$$

(8) $$\Delta =\omega_{p}^{2}\gamma \left[{\left(\omega_{T}^{2}-\omega^{2}\right)}^{2}+{\left(\omega \eta \right)}^{2}\right]+\omega^{2}\eta \left(\omega_{L}^{2}-\omega_{T}^{2}\right)\left(\omega^{2}+\gamma^{2}\right)$$

and $\omega_{L}$ is the longitudinal optical mode frequency; $\omega_{T}$ is the transverse optical mode frequency; *η* is the phonon damping constant; γ is the plasma damping constant; *n*_1_ and *n*_2_ are refractive indices at incident frequency *ω*_1_ and scattering frequency *ω*_2_, respectively; C is the Faust–Henry coefficient, where the value here is about 0.35; *α* is the polarizability; *E* is the macroscopic electric field; and *n_ω_* is the Bose–Einstein factor. The dielectric function is described as

(9) $$\varepsilon =\varepsilon_{\infty }\left(1+\frac{\omega_{L}^{2}-\omega_{T}^{2}}{\omega_{T}^{2}-\omega^{2}-i\omega \eta }-\frac{\omega_{p}^{2}}{\omega \left(\omega +i\gamma \right)}\right)$$

(10) $$\omega_{p}^{2}=\frac{4\pi ne^{2}}{\varepsilon_{\infty }m^{*}}$$

where *ω_p_* is the plasma frequency; *n* is the free carrier concentration; m* is the effective mass and e is the unit charge; and *ε_∞_* is a high-frequency dielectric constant. For polar semiconductors, strong coupling between the LO phonon and the free carrier plasmon exists. By taking C (above) and other fitting parameters, the line shape of the LO phonon–plasmon coupled mode can be fitted by means of the least square difference. This method has been successfully applied to obtain the carrier concentrations in different semiconductors [32].

**Figure 16:**
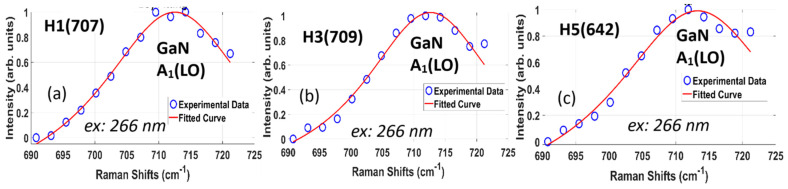
DUV 266 nm excitation Raman spectra of GaN A_1_(LO) modes and fittings from three AlGaN-GaN HEMTs, (**a**) for H1(707), (**b**) for H3(709) and (**c**) for H5(642), respectively, with open circles for experimental data and red solid lines for calculated results.

Figure 16 shows the experimental and fitted DUV 266 nm Raman spectra of GaN A_1_(LO) modes from three AlGaN-GaN HEMTs, with the GaN A_1_(LO) peak, FWHM, and calculated parameters listed in Table 7.

**Figure 17:**
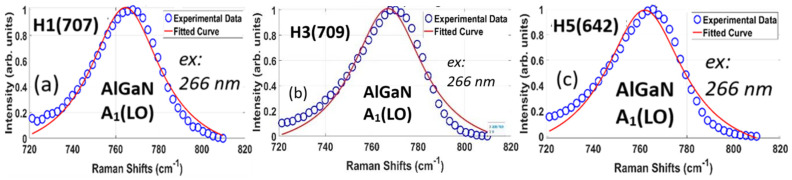
Experimental and fitted DUV 266 nm excitation Raman spectra of AlGaN A_1_(LO) modes from three AlGaN-GaN HEMTs of (**a**) H1(707), (**b**) H3(709), and (**c**) H5(642), with AlGaN A_1_(LO) peak, FWHM, and calculated parameters listed in Table 8.

Figure 17 shows the experimental (open circles) and fitted DUV 266 nm Raman spectral data of the AlGaN A_1_(LO) modes and fittings (red solid lines) from three AlGaN-GaN HEMTs, with the AlGaN A_1_(LO) peak, FWHM, and calculated parameters listed in Table 8.

From Figure 13, the AlGaN A1(LO) peaks for the two samples of 707 and 639, i.e., H1 and H2, are almost matched together, and those for 641 (H4) and 642 (H5) exhibit the two peaks at the same frequency. Therefore, H1 (707) and H2 (639) should have the same value of the carrier concentration, and so do for the pair of H4 (641) and H5 (642). We choose only one of each pair to perform the calculation, i.e., with only H1 (707) and H5 (642) (see Figure 16 and Table 8), together with H3 (709).

The results in Table 3 show that H3 (709) has an AlGaN A1(LO) peak frequency and calculated carrier concentration higher than other samples. These indicate the variation in carrier concentration in the AlGaN layer in correlation with the thickness of the AlGaN layer. As the d(AlGaN) increases from 13 nm and 26 nm to 39 nm, the carrier concentration in the AlGaN layer increases to a maximum value, then decreases as d(AlGaN) increases to 65 nm and 103 nm. This means an optimized AlGaN layer thickness is obtained. Also, the fitting accuracies indicated in Table 8 are beyond 87%, which is quite good. The differences between the experimental and simulation data are partially from the effects of the GaN A1(LO) mode near the left wings of the AlGaN A1(LO) mode.

## 4. Conclusions

In summary, a series of AlGaN-GaN high-electron-mobility transistor (HEMT) structures grown by MOCVD on c-sapphire, consisting of a GaN thick layer (~1.6 μm) and Al_x_Ga_1−x_N (x~0.25) film on top with a variable thickness of 13–104 nm, are investigated by multiple techniques, including high-resolution X-ray diffraction (HR-XRD), DUV (266 nm), and UV (325 nm) excitation photoluminescence (PL) and Raman scattering, and variable-angle spectroscopic ellipsometry (VASE) and temperature-dependent (TD) SE. Our AlGaN-GaN HEMTs are improved with high crystalline structures. The HRXRD and calculations exhibited the extremely low screw dislocation density of 1.6 × 10^8^ cm^−2^, superior to many results from literature. Comparative PL under 266/325 nm excitations reveals and confirms the 2DEG and influences within HEMTs. The DUV (266 nm) Raman and calculations based upon the LOPC simulation determined the carrier concentrations in compatible AlGaN and GaN, and the special correlation model (SCM) simulations on the E_2_(high) modes revealed that the whole 1.6 μm thick GaN layer was under compressive stress, while the GaN top area near the AlGaN layer was under tensile stress. VASE measurements and analyses precisely obtained the layer thicknesses of HEMT structures, optical constants of n & k and absorption coefficients, energy bandgaps, Urbach energy values (E_U_), and temperature relationships of Eg~T. Three methods employed in determining the energy bandgaps, i.e., n(max), the (αhν)^2^ ~ eV relationship, and the α ~ eV relationship, are compared. It is concluded that two simple ways, i.e., via n(max) and the α ~ eV relationship, are good for primary/quick estimation of bandgaps, appliable in industry production environments. In comparison with other research works on AlGaN-GaN HEMTs in the literature [6,7,8,9,10,11,12,13,14,15,16,17,18,19,22,23,24,25,26,27,28], our studies provide unique results and new contributions to the field. For example, spectroscopic ellipsometry (SE) and variable-temperature SE studies on AlGaN-GaN HEMTs are scarcely seen in the literature. AlGaN-GaN HEMT structures with variable AlGaN layer thicknesses and effects are also not common in the abovementioned literature. Our studies and unique results should provide useful references for the R&D of HEMT and related structures/devices.

## Figures and Tables

**Table 1:** Information for AlGaN-GaN HEMT samples, obtained from VASE analyses.

Sample Name	H1(707)	H2(639)	H3(709)	H4(641)	H5(642)
Surface roughness by SE (nm)	3.0	1.4	1.9	1.3	1.4
Thickness of AlGaN by SE (nm)	15.6	25.2	38.0	66.6	106.0
Thickness of GaN by SE (nm)	1556.4	1607.6	1552.9	1600.4	1593.4
Thickness of AlN by SE (nm)	23.9	24.4	22.8	23.4	24.9
MSE	9.94	10.64	10.27	9.33	9.76
λ (n_max_) of AlGaN (nm)	300.4	306.1	300.4	305.9	305.8
E_g_ (from n_max_) of AlGaN (eV)	4.128	4.051	4.128	4.054	4.055
λ (n_max_) of GaN (nm)	362.5	362.5	362.2	362.3	362.5
E_g_ (from n_max_) of GaN (eV)	3.421	3.421	3.424	3.424	3.421

**Table 2:** Peak/FWHM and calculated results of screw dislocation density of five GaN films.

Sample Name	H1(707)	H2(639)	H3(709)	H4(641)	H5(642)
Peak 2θ (0002) (°)	34.45	34.46	34.47	34.45	34.45
FWHM 2θ (0002) (°)	0.136	0.135	0.150	0.084	0.120
β: (2θ_FWHM_*π/180, Rad)	2.268 × 10^−3^	2.268 × 10^−3^	2.617 × 10^−3^	1.340 × 10^−3^	2.093 × 10^−3^
β^2^	5.143 × 10^−6^	5.143 × 10^−6^	5.843 × 10^−6^	1.870 × 10^−6^	4.381 × 10^−6^
*D_screw_* (×10^8^) (cm^−2^)	4.39	4.39	5.00	1.60	3.74

Note: b = 0.5185 nm = 0.5185 × 10^−7^ cm; b^2^ = 0.2688 × 10^−14^ cm^2^; 4.36b^2^ = 1.172 × 10^−14^ cm^2^.

**Table 3:** 2θ peak/FWHM and calculated results of screw dislocation density of three AlGaN films.

Sample Name	H3(709)	H4(641)	H5(642)
Peak 2θ (0002) (°)	35.03	34.97	34.98
FWHM 2θ (0002) (°)	0.320	0.141	0.132
β: (*π/180, Rad)	5.582 × 10^−3^	2.440 × 10^−3^	2.268 × 10^−3^
β^2^	31.16 × 10^−6^	5.96 × 10^−6^	5.143 × 10^−6^
*D_screw_* (×10^8^) (cm^−2^)	28.22	6.93	4.66

**Table 4:** Values from Lorentz fit on AlGaN and GaN PL spectra (ex: 266 nm), including GaN PL sideband emissions.

Sample Name	H1(707)	H2(639)	H3(709)	H4(641)	H5(642)
AlGaN PL peak (eV)	4.100	4.043	4.109	4.051	4.059
FWHM of AlGaN PL peak (meV)	111	96	109	89	87
GaN_PL_ main peak (eV)	3.445	3.444	3.446	3.444	3.445
FWHM of AlGaN PL peak (meV)	32	30	25	24	27
GaN_sidePL1_ peak (eV)	3.407	3.410	3.426	3.425	3.419
FWHM-GaN_sidePL_ peak (meV)	32	30	24	24	27
GaN_sidePL2_ peak (eV)	3.370	3.375	3.400	3.400	3.389
FWHM-GaN_sidePL_ peak (meV)	29	29	24	24	27

**Table 5:** E2(high) peak, FWHM, and calculated parameters based upon the spatial correlation model (SCM).

Sample Name	H1(707)	H2(639)	H3(709)	H4(641)	H5(642)
A (cm^−1^)	546.0	546.5	546.5	545.0	544.5
B (cm^−1^)	100	108	102	108	109
L (Å)	19	21	20	27	26
Γ_0_ (cm^−1^)	11	14	16	18	21

**Table 6:** GaN E2(high) peak, and calculated parameters for five AlGaN-GaN HEMTs.

Sample Name	H1(707)	H2(639)	H3(709)	H4(641)	H5(642)
A (cm^−1^)	571.2	571.0	571.1	570.6	571.1
B (cm^−1^)	100	106	102	103	107
L (Å)	27	29	30	31	37
Γ_0_ (cm^−1^)	2.5	2.6	2.9	3	3.1

**Table 7:** GaN A1(LO) peak, FWHM, and calculated parameters.

Sample Name	H1(707, 13 nm)	H3(709, 39 nm)	H5(642, 104 nm)
Peak Position (cm^−1^)	712.5	712.7	713.3
FWHM (cm^−1^)	29.0	26.4	31.0
Phonon Lifetime (ps)	0.18	0.20	0.17
Fitting Accuracy	95.8%	94.2%	93.4%
Plasmon Frequency (THz)	0.13	0.13	0.13
Plasmon Damping Constant (THz)	5.47	4.98	5.84
Carrier Concentration (×10^18^ cm^−3^)	1.13	1.13	1.14

**Table 8:** AlGaN A1(LO) peak, FWHM, and calculated parameters.

Sample Name	H1(707, 13 nm)	H3(709, 39 nm)	H5(642, 104 nm)
Peak Position (cm^−1^)	764.5	767.0	762.0
FWHM (cm^−1^)	40.2	37.6	40.8
Phonon Lifetime (ps)	0.13	0.14	0.13
Fitting Accuracy	90.6%	87.3%	88.0%
Plasmon Frequency (THz)	0.14	0.15	0.14
Plasmon Damping Constant (THz)	7.6	7.1	7.7
Carrier Concentration (×10^18^ cm^−3^)	1.31	1.31	1.30

## Data Availability

The data presented in this study are available on request from the corresponding author.

## Supplementary Materials

The following supporting information can be downloaded at: https://www.mdpi.com/article/10.3390/nano15030165/s1.
