# Supplementary material for: Spectroscopic Ellipsometry and Correlated Studies of AlGaN-GaN HEMTs Prepared by MOCVD
Source: Nanomaterials (Basel). 2025 Jan 22;15(3):165. doi: 10.3390/nano15030165 (PMC11820650; doi:10.3390/nano15030165)
Supplement: Supplementary file 1 [file nanomaterials-15-00165-s001.zip › nanomaterials-3384330-supplementary.pdf]

# Supplementary Materials figure S1 and figure S2

## Figure S1:

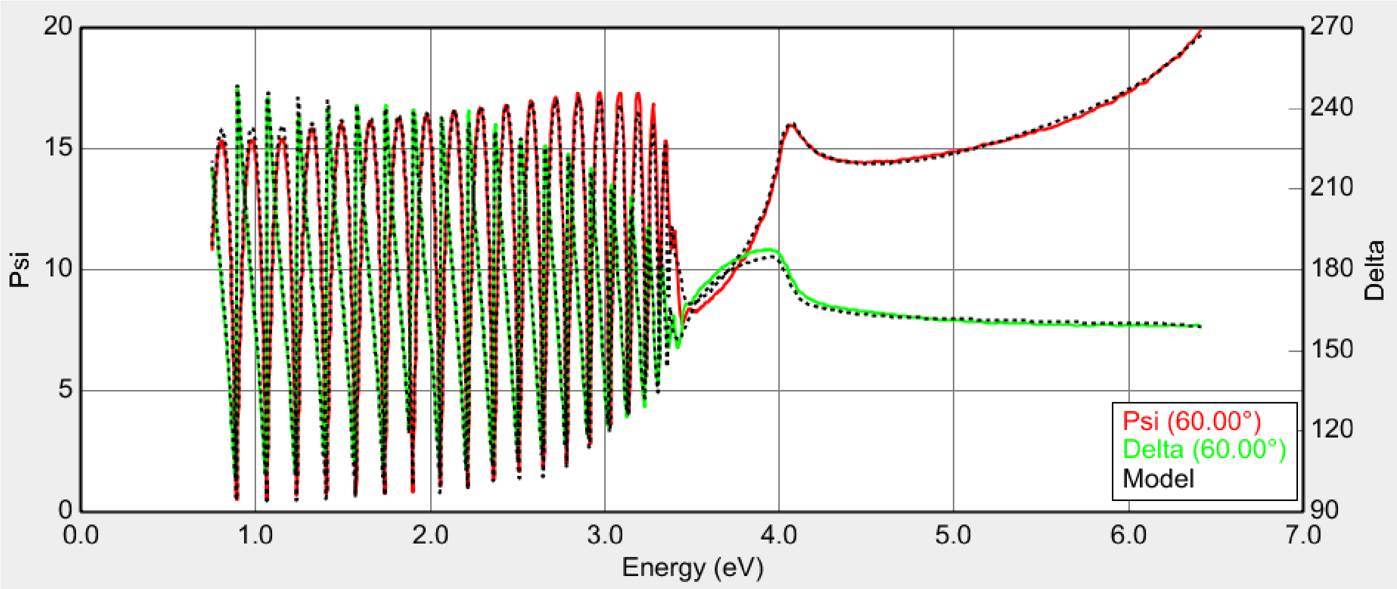


**Figure S1 (a) H2(639)**

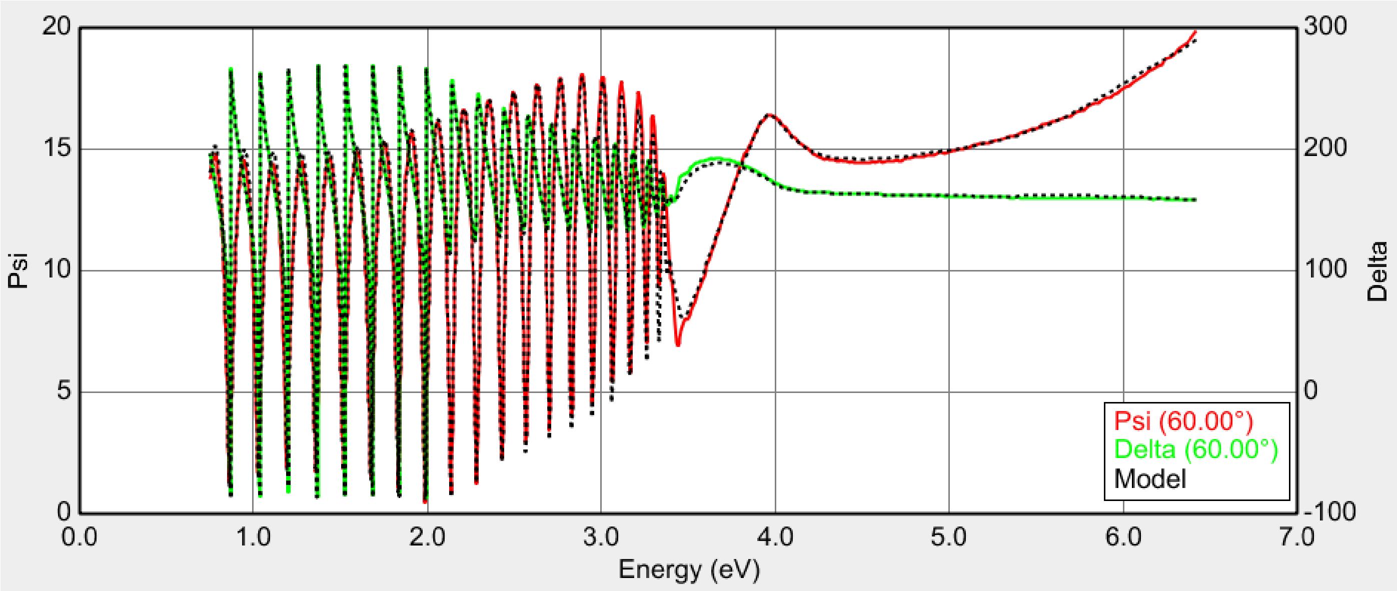


**Figure S1 (b) H5(642)**

*Additional plots of SE Psi and Delta spectra for* H1(707), H3(709) and H4(641)*:*

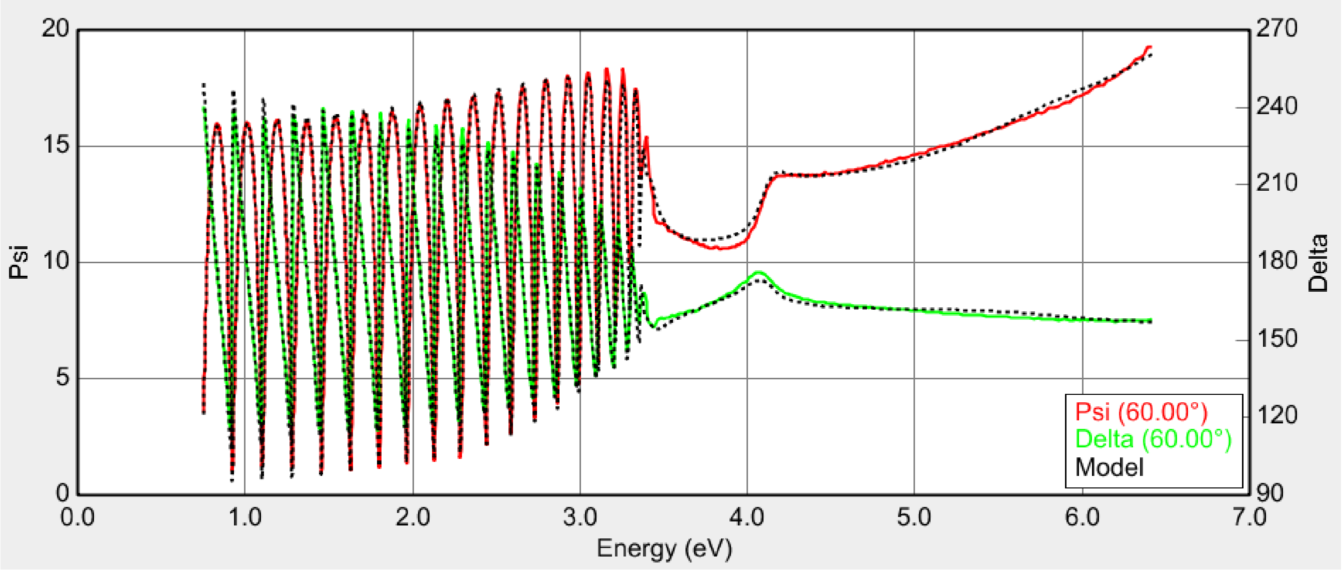


**H1(707)**

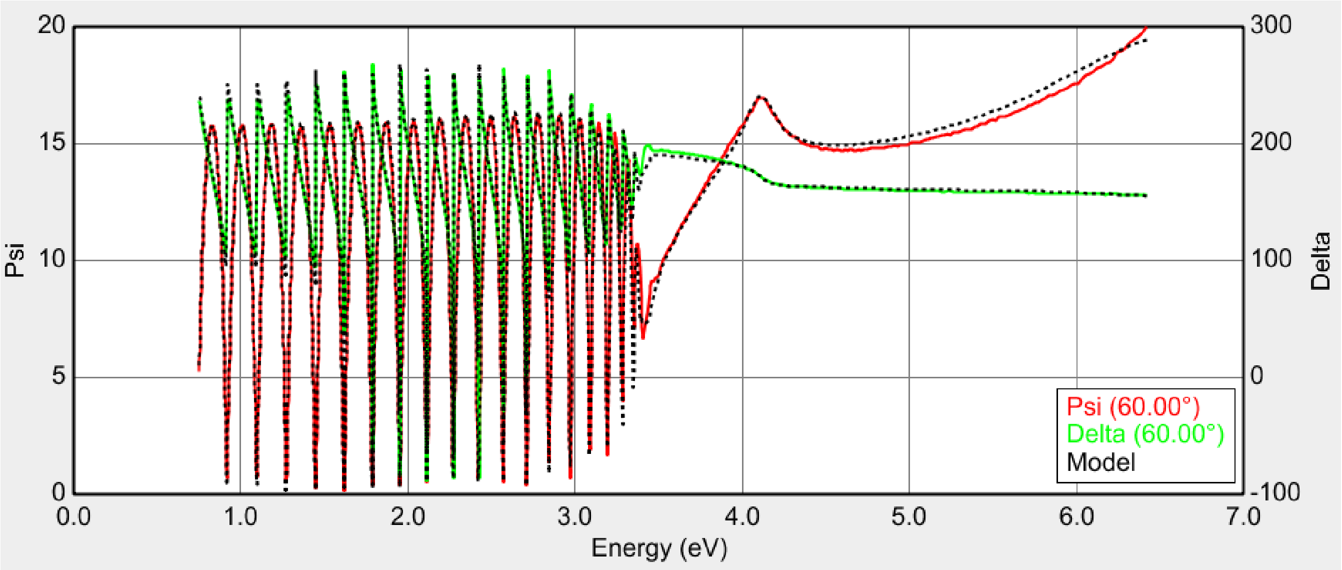


**H3(709)**

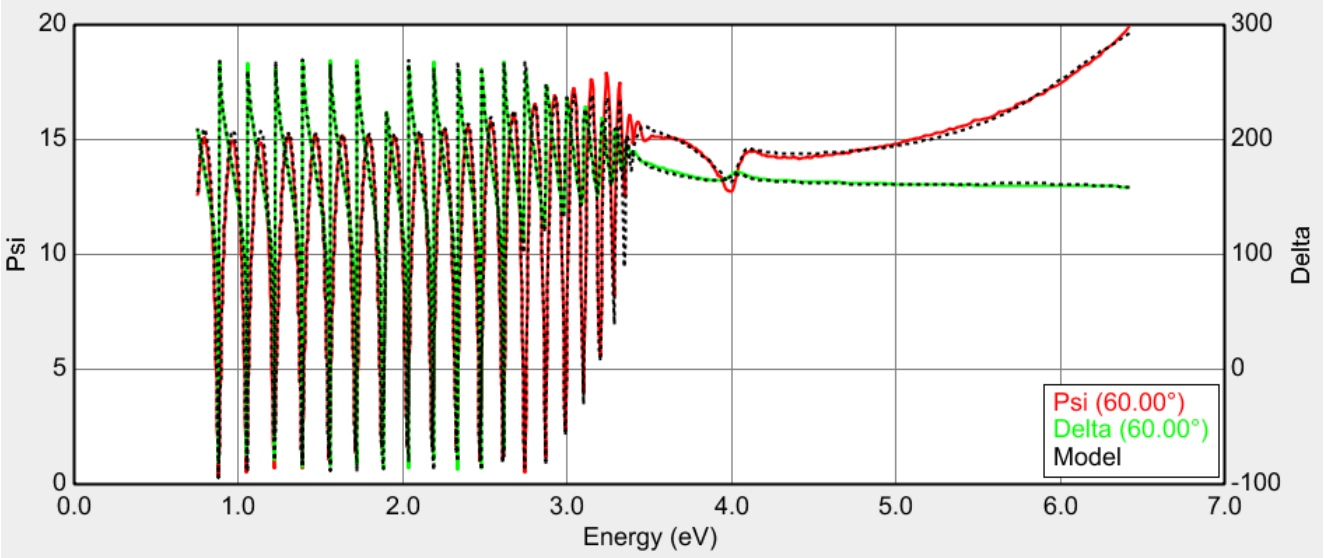


**H4(641)**

# figure S2:

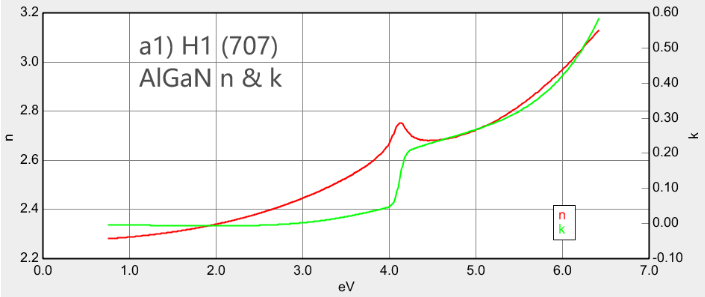


**(a1) H1 (707) AlGaN n & k**

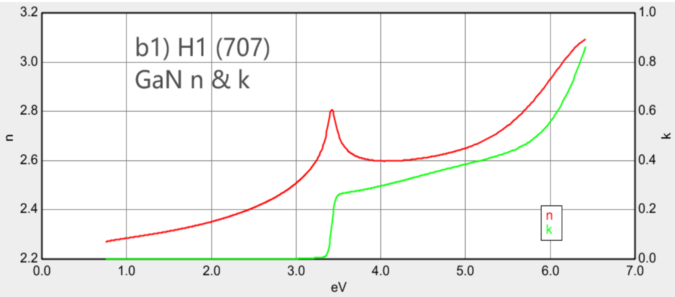


**(b1) H1 (707) GaN n & k**

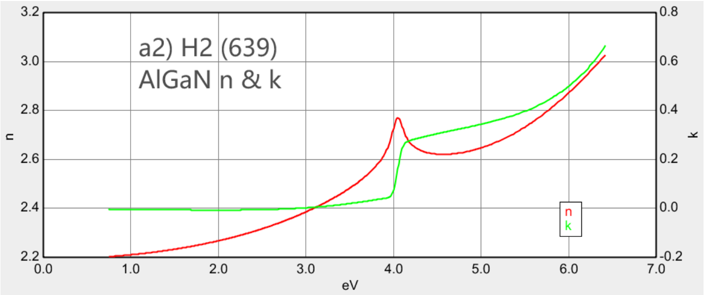


**(a2) H2 (639) AlGaN n & k**

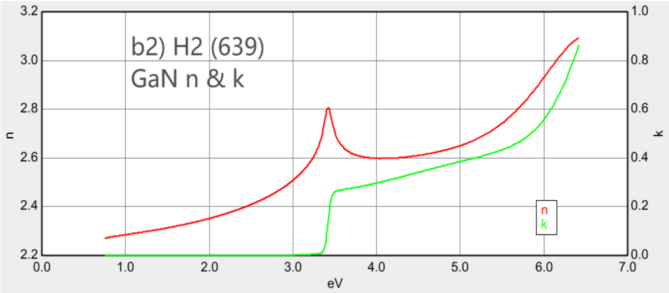


**(b2) H2 (639) GaN n & k**

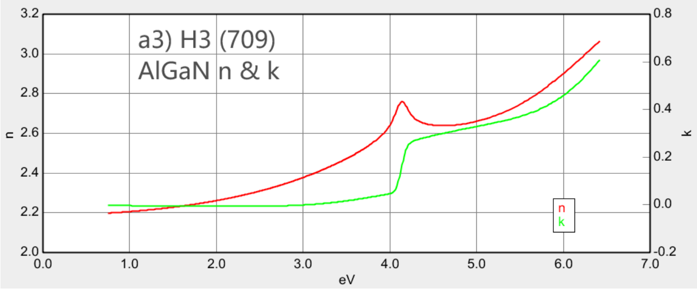


**(a3) H3 (709) AlGaN n & k**

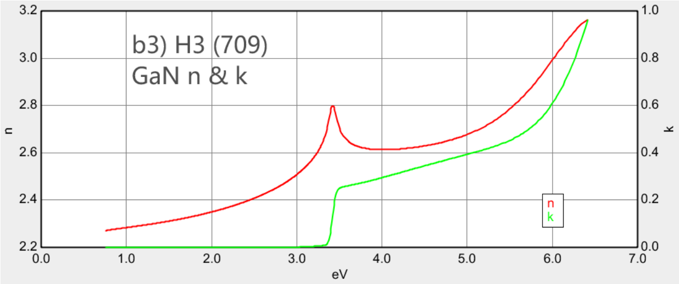


**(b3) H3 (709) GaN n & k**

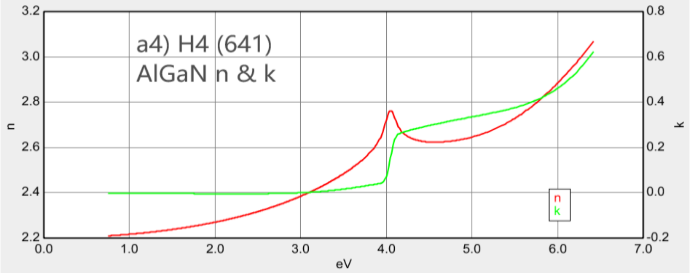


**(a4) H4 (641) AlGaN n & k**

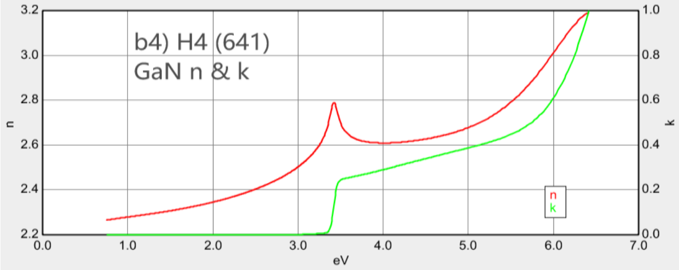


**(b4) H4 (641) GaN n & k**

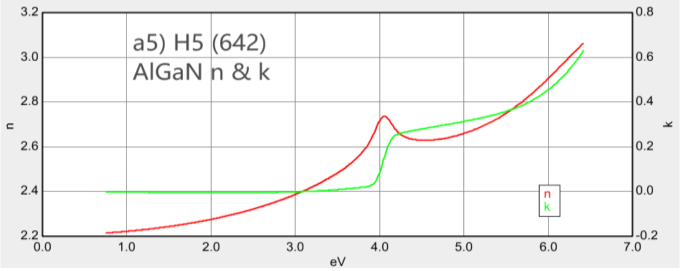


**(a5) H5 (642) AlGaN n & k**

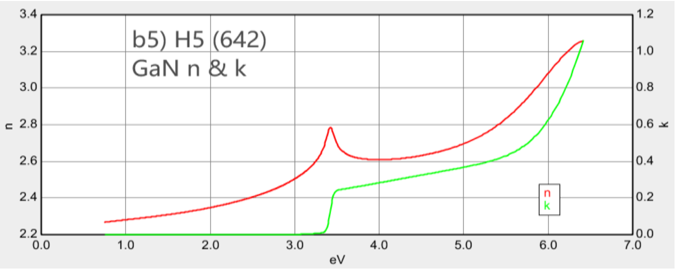


**(b5) H5 (642) GaN n & k:**
